# Delayed Diagnosis of Intestinal Tuberculosis: A Case Report

**DOI:** 10.7759/cureus.30600

**Published:** 2022-10-23

**Authors:** Tiago Araújo, Ana Silva, Pedro Laranjo, Yulia Shigaeva, Teresa Bernardo

**Affiliations:** 1 Internal Medicine Department, Unidade Local de Saúde do Litoral Alentejano, Santiago do Cacém, PRT

**Keywords:** pulmonary tuberculosis, mycobacterium tuberculosis, ileocecal tuberculosis, intestinal tuberculosis, abdominal tuberculosis, extrapulmonary tuberculosis

## Abstract

Abdominal tuberculosis is an uncommon clinical entity, and it can involve the gastrointestinal tract but also the peritoneum, lymph nodes, and solid organs. Its prevalence is higher among individuals from endemic regions for tuberculosis. Epidemiological risk factors associated with typical symptoms and complementary exams should prompt early treatment.

We describe the case of a 47-year-old man, originally from India, residing in Portugal for approximately a year. He presented to our emergency department with a three-week-long history of diarrhea, diffuse abdominal pain, more intense on the left quadrants of the abdomen, anorexia, asthenia, and loss of nearly 10% of his body weight. Abdominal and pelvic imaging showed diffuse circumferential thickening of the distal ileum and adjacent mesentery with associated lymphadenopathies. A colonoscopy confirmed the presence of an ulcerated deformative lesion of the cecum with the involvement of the terminal ileum. Initial suspicion of infectious colitis versus inflammatory bowel disease led the team to prescribe antibiotics and corticosteroid therapy, which was associated with bronchoalveolar lavage and sputum samples negative for *Mycobacterium tuberculosis*, delaying the diagnosis of intestinal tuberculosis. The lack of improvement after weeks of the initial medical therapy, and with histopathological examination of cervical lymphadenopathy showing the presence of granulomatous lymphadenitis with necrosis, led the medical team to start antituberculostatic therapy. The patient showed significant clinical and laboratory improvement, but after two months of adequate treatment a cavitated nodule appeared on the upper lobe of the left lung, and a *Mycobacterium tuberculosis* *complex* was identified in the bronchoalveolar lavage.

Timely diagnosis and adequate treatment are essential to lower mortality rates of intestinal tuberculosis, and epidemiological risk factors have a great deal of importance on this matter and must always be taken into account.

## Introduction

Tuberculosis (TB) is an infectious disease caused by *Mycobacterium tuberculosis* that typically affects the lung tissue (pulmonary TB) but can also affect other sites (extrapulmonary TB) [1]. Nearly a quarter of the world’s population has been infected with *Mycobacterium tuberculosis*, even though only a relatively small percentage will develop TB disease in their lifetime. Conversely, there is a higher probability of developing TB disease among individuals living with Human Immunodeficiency Virus (HIV) and those affected by other risk factors, including malnutrition, diabetes, smoking, and alcohol consumption [2]. TB can affect anyone in any part of the world, still endemic in many countries. The vast majority of patients are adults, with more cases among men than women [2]. TB is the 13^th^ leading cause of death worldwide, with 1.5 million deaths in 2020 [1]. Extrapulmonary TB accounts for about 20% of patients with TB, of which only 10% present with intestinal TB [1]. Diagnosing intestinal tuberculosis can be challenging since it mimics symptoms of many other intestinal pathologies. Therefore, epidemiological risk factors are extremely important in arising clinical suspicion. Treatment is similar to pulmonary TB; however, mortality rates are variable, ranging from 1.4%-20%, depending on a number of clinical risk factors [1,3-5].

## Case presentation

A 47-year-old-man, originally from India and living in Portugal for approximately a year, without previously known diseases or risk factors for tuberculosis, presented to our emergency department (ED) with a three-week-long history of diarrhea, without blood, mucus, or pus, diffuse abdominal pain (intensity 7/10), more intense on the left quadrants, abdominal distension, nonselective anorexia, asthenia and loss of 5.5kg (approximately 10% of his body weight). He denied fever, medication intake, or ingestion of raw meat or unpasteurized dairy products.

On physical examination, the patient presented pale mucous membranes, and a distended abdomen, with diffuse mild pain on superficial palpation, more intense on the left quadrants on deep palpation, without areas of rigidity but with guarding. Murphy and Blumberg's signs were absent, and no abdominal mass was palpable. Abdominal tympany was diffusely increased, and bowel sounds were hypoactive without changes in pitch.

**Figure 1:**
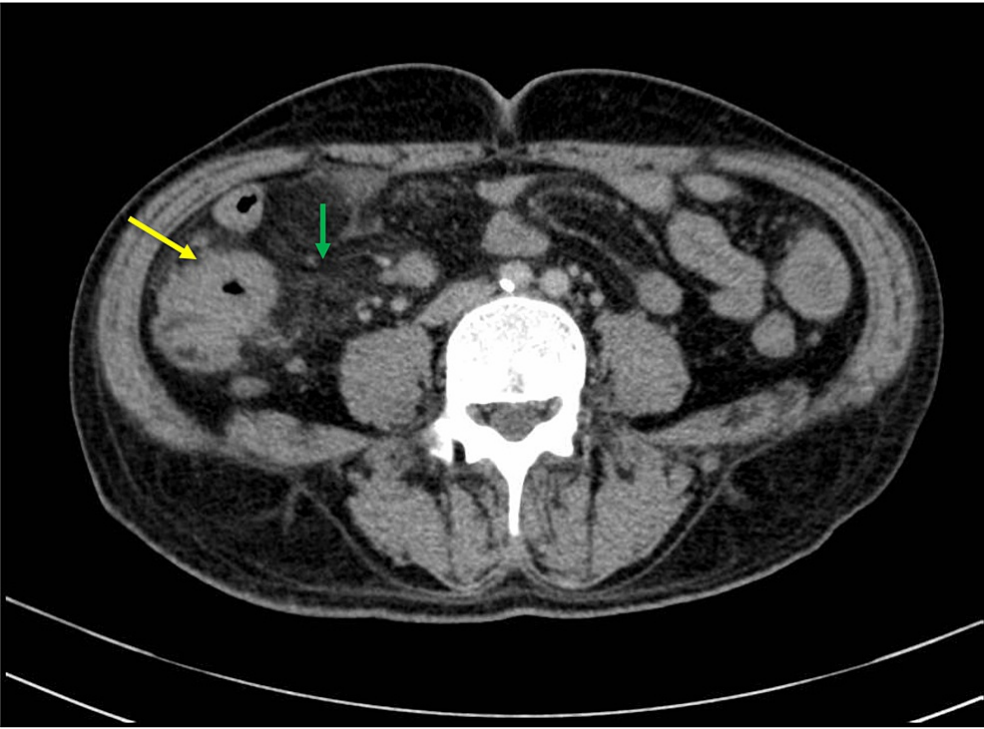
Contrast-enhanced abdominal-pelvic computed tomography. Thickening of the cecum (yellow arrow) and adjacent mesentery (green arrow).

Abdominal radiography was normal and laboratory data showed microcytic and hypochromic anemia with hemoglobin of 9.1 g/dL (normal range 12.0-17.0 g/dL), thrombocytosis with a platelet count of 549x10^9^/L (normal range 150-450x10^9^/L), increased C-reactive protein of 17.2 mg/dL (normal range <1.0 mg/dL), with normal blood cell count, no increase in hepatic or cholestatic enzymes and normal urinalysis. A contrast-enhanced abdominal and pelvic computed tomography (CT) was performed, showing diffuse circumferential thickening of the distal ileum and adjacent mesentery, with associated lymphadenopathies suggestive of inflammatory or infectious disease; although an intestinal neoplasm was also considered (Figure 1).

The patient was admitted so that a colonoscopy could be performed to confirm the diagnosis. Feces and blood cultures were performed at that time, but no antibiotic therapy was started since the patient was hemodynamically stable, and there was no certainty that the cause was a bacterial infection. For the next four days the patient had daily fever without predominance in time of day, associated with an increase in C-reactive protein levels (18.2 mg/dL), without leukocytosis or neutrophilia. Additional bloodwork showed a severe iron deficiency with low serum iron of 5.0 mcg/dL (normal range 45-182 mcg/dL), normal Ferritin of 224 ng/mL and low Transferrin of 77 mg/dL (normal range 180-329 mg/dL), elevated erythrocyte sedimentation rate (ESR) of 74 mm/hr (normal range 0-20mm/hr), severe hypoproteinemia with 1.5 g/dL of albumin (normal range 3.5-5.2 g/dL), negative anti-neutrophil cytoplasmic antibodies, negative anti-saccharomyces cerevisiae antibody and negative anti-nuclear antibodies.

**Figure 2:**
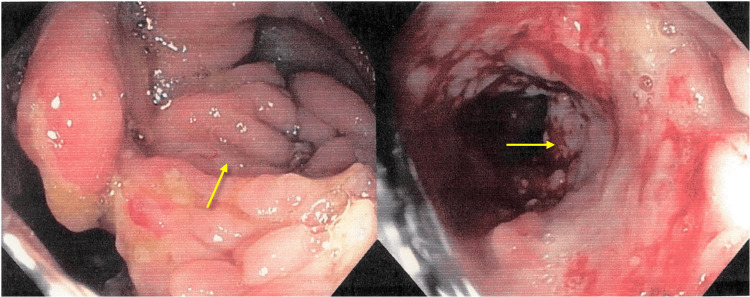
Total colonoscopy. Ulcerated deformative lesion of the cecum, with involvement of the terminal ileum (yellow arrows).

Due to the persistence of fever, abdominal pain, diarrhea, and the increase of inflammatory markers, antibiotic therapy was started with Levofloxacin 750 mg daily. Three days after, the patient showed no signs of improvement and had a daily fever, abdominal pain, and a progressive increase of C-reactive protein (28.3 mg/dL). Feces cultures were negative for *Shigella spp.*, *Salmonella spp.*, *Campylobacter spp.*, *Yersinia enterocolitica*, *Entamoeba histolytica*, *Giardia lamblia*, *Cryptosporidium parvum*, *Staphylococcus aureus,* and *Clostridioides difficile,* and blood cultures were also negative. A colonoscopy was performed on the seventh day after admission and showed an ulcerated deformative lesion of the cecum with involvement of the terminal ileum, macroscopically suggestive of Crohn’s Disease versus intestinal neoplasm (Figure 2).

Biopsies were performed, and the Gastroenterology team suggested switching antibiotic therapy to a combination of Ciprofloxacin 400 mg every 12 hours, Metronidazole 500 mg every eight hours, and also starting treatment with Mesalazine 1000 mg every eight hours, suspicion of inflammatory bowel disease with a superimposed bacterial infection. For the next 10 days, the patient showed improvement in the intensity of the abdominal pain (3/10) associated with cessation of diarrhea and a significant decrease in the C-reactive protein levels (6.9 mg/dL); however, having a daily fever. On the 17^th^ day after admission, the results of intestinal biopsies revealed no malignant cells, with almost the entire sample constituted of granulation tissue with just one fragment of colonic mucosa showing erosion, suggestive of ulcer base and edge. The results were again discussed with the Gastroenterology team and the patient was started on systemic corticosteroid therapy with Prednisolone 1mg/kg based on the main suspicion of unspecified inflammatory bowel disease. Previously mentioned antibiotic therapy was also continued until 21 days were completed.

After 13 days of corticosteroid therapy, the patient kept having daily fever, C-reactive protein levels increased again (16.6 mg/dl), and he presented with *de novo* cervical lymphadenopathies. That, associated with the absence of significant improvement in the patient's condition after having completed a total of 24 days of antibiotic therapy and 13 days of corticosteroid therapy, led the medical team to suspect other etiologies, particularly intestinal tuberculosis. Subsequently, additional bloodwork was performed, including an Interferon Gamma Release Assay (IGRA), antibody testing for B and C hepatitis virus (HBV and HCV) and Human Immunodeficiency Virus (HIV), identification of *Mycobacterium tuberculosis complex* (MTC) by real-time polymerase chain reaction (RT-PCR) on a sputum sample, the bronchoscopy with broncho-alveolar lavage with bacteriologic, mycobacterial, mycologic examinations and a new contrast-enhanced cervical, thoracic, abdominal and pelvic CT. Additionally, one of the cervical lymphadenopathies was removed and sent to anatomopathological, bacteriologic, mycobacterial, and mycologic examinations.

**Figure 3:**
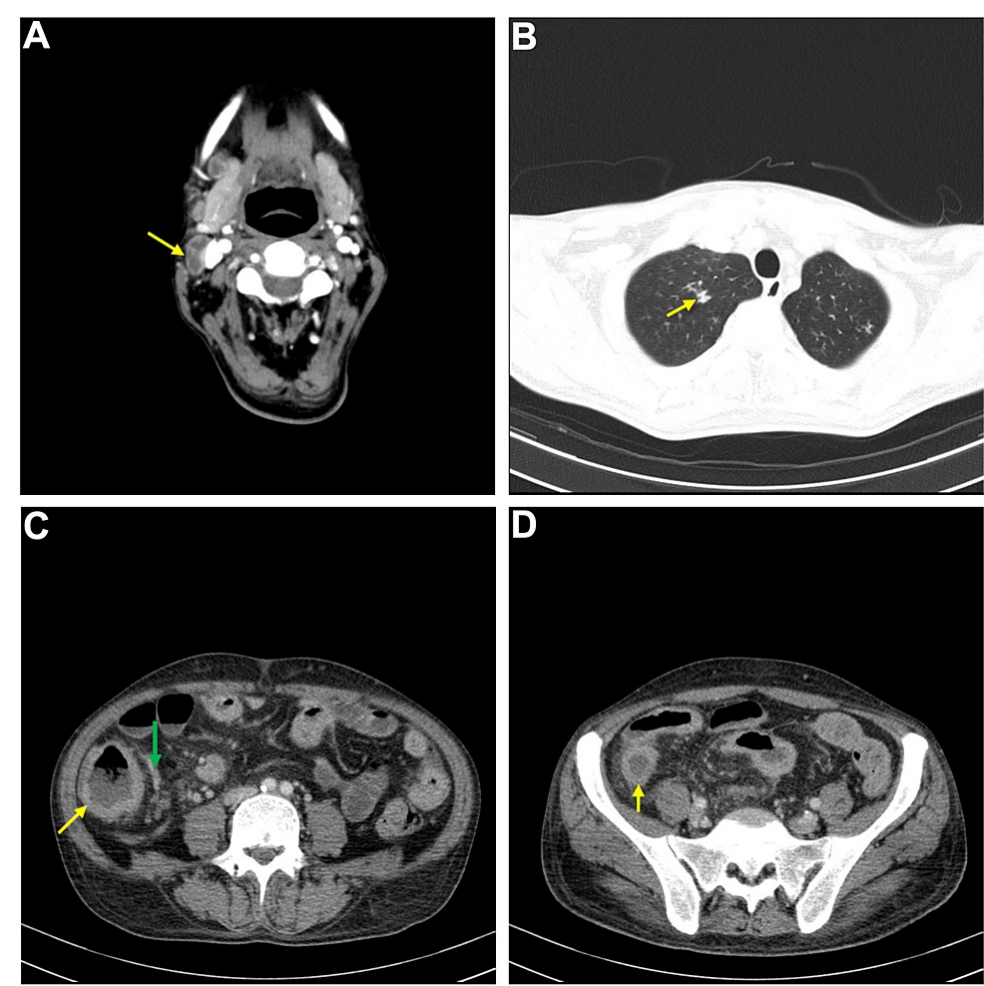
Contrast-enhanced cervical, thoracic, abdominal, and pelvic computed tomography. A: Jugular lymphadenopathy (yellow arrow); B: Small areas of fibrotic densification with traction bronchiectasis on the upper lobes (yellow arrow); C: Severe thickening of the ascending colon (yellow arrow) and adjacent mesentery (green arrow); D: Thickening of the cecum (yellow arrow).

The CT showed multiple cervical necrotic lymphadenopathies, small areas of fibrotic densification associated with small nodules on both lungs, and worsening of the intestinal involvement, with an extensive concentric infiltrative thickening of the cecum, ascending colon, and terminal ileum and the adjacent mesentery and associated with regional lymphadenopathies (Figure 3).

Based on the clinical course of the disease and the CT findings, the patient was started on antituberculostatic empiric therapy with Isoniazid 300 mg daily, Rifampicin 600 mg daily, Ethambutol 1200 mg daily and Pyrazinamide 1500 mg daily, and also on a corticosteroid tapering regimen. The RT-PCR for MTC in the sputum sample was negative, the bronchoscopy was normal, and the bronchoalveolar lavage examinations were irrelevant. IGRA and HCV antibody testing were both positive. The result of the anatomopathological examination of the cervical lymphadenopathy showed a necrotic central area highly suggestive of granulomatous lymphadenitis of possible mycobacterial etiology; however, the sample was insufficient for mycobacterial examination.

In the next days and weeks, the patient showed significant improvement in his condition, with complete resolution of abdominal pain and normalization of all inflammatory markers, including an ESR of 16 mm/hr. He was discharged after 60 days of admission, asymptomatic, with instructions to complete at least six months of antituberculostatic therapy. Viral load and genotype evaluation of HCV were also performed, and the patient was referred to the Infectious Diseases consultation for treatment and monitoring. The abdominal ultrasound was normal. 

Approximately two months after discharge, the patient was admitted to another hospital because of toxic hepatitis due to antituberculostatic agents, prompting treatment interruption. The patient denied alcohol consumption and wasn’t taking any other hepatotoxic medication. Serum iron and ferritin were normal, excluding hemochromatosis. Antinuclear antibodies, anti-smooth muscle antibodies, anti-liver/kidney microsomal antibodies, serum ceruloplasmin levels, alpha-1 antitrypsin levels, and thyroid hormones were all normal. Abdominal ultrasound was normal, but liver elastography showed severe liver scarring (fibrosis score F3). A new contrast-enhanced thoracic, abdominal, and pelvic CT was performed, showing a normal liver, with significant improvement of intestinal findings, with only mild thickening of the terminal ileum and ileocecal valve. However, it showed a small cavitated nodule in the superior lobe of the left lung. A new bronchoscopy was performed, and it was possible to identify MTB by RT-PCR in the bronchoalveolar lavage. After cessation of antituberculostatic therapy, normalization of hepatic enzymes was achieved. After six weeks, antituberculostatic therapy was restarted, with the same drugs, without new evidence of toxicity. During this time, the patient remained completely asymptomatic. He was again discharged with instructions to continue antituberculostatic therapy and with a scheduled Infectious Diseases appointment to start treatment for HCV infection. However, the patient decided to return to India shortly after discharge, and it was impossible to monitor his compliance with therapy and clinical status.

## Discussion

As mentioned previously, intestinal TB is not a common presentation of extrapulmonary TB. In its presentation spectrum, two types of bowel lesions can be seen: ulcerative and ulcerohypertrophic. The first one has been mainly described in malnourished individuals; on the other side, the latter has been described in relatively well-nourished individuals. Ulcerative and stricturous forms more commonly affect the small intestine, while colonic and ileocecal forms are usually ulcerohypertrophic [6]. The most common site of involvement is ileocecal region (25%-90%), followed by small intestine (6%-67%), colon (2%-32%), and gastroduodenal area (0.5%-5%) [6].

Intestinal TB should be suspected in patients with clinical manifestations and relevant epidemiological factors (such as a prior known tuberculosis infection, possible tuberculosis exposure, and/or past or present residence in, or travel to, an area with endemic tuberculosis) [1]. Other risk factors for intestinal tuberculosis include cirrhosis, HIV infection, diabetes mellitus, underlying malignancy, malnutrition, treatment with antitumor necrosis factor agents, corticosteroids, and use of continuous ambulatory peritoneal dialysis [6].

In terms of clinical presentation, the signs and symptoms associated with intestinal TB are nonspecific and easily confused with other intestinal diseases [1]. The most common clinical manifestations are abdominal pain (76%-88%), weight loss (50%-80%) and fever (43%-80%) [1,7,8]. Abdominal pain can be acute or chronic and, in some cases, acute on chronic when associated with complications [1,8]. The abdominal pain is typically located in the lower right quadrant and the periumbilical region [1,7,8]. Weight loss in patients with intestinal tuberculosis is multifactorial, being associated with the chronic inflammatory process, decreased contribution, and changes in intestinal absorption [1,8]. The fever is commonly irregular and low-grade (37.5ºC to 38.5ºC) and is associated with night sweats [1,7,8]. Other gastrointestinal symptoms that may occur include diarrhea, nausea, vomiting, and constipation [1,7,8]. On physical examination, ascites (10%-34%), palpable abdominal mass (10%-17%), and splenomegaly (14.2%) may be found [1,7,8]. Multiple areas of the gastrointestinal tract may be affected, the most common being the ileocecal region, present in more than 75% of patients [8]. Furthermore, patients with intestinal tuberculosis may also present as asymptomatic [1]. Despite being unspecific for intestinal TB, our patient presented with some of the most common symptoms of this pathology. However, because he was living in Portugal for more than a year, the fact that he came from India was wrongly overlooked, contributing to the delay in diagnosis.

The definite diagnosis of intestinal tuberculosis is challenging and based on microbiological, histopathological, immunological, and imaging studies. The acid-fast bacilli (AFB) staining has very high specificity (100%) [1]. However, it has a low sensitivity (17%-31%), so the risk of false negatives is very high [1]. The gold standard test for diagnosis of intestinal tuberculosis is *Mycobacterium tuberculosis* culture [1]. It is very specific (100%), although it has a very low sensitivity value (9.3%) [1]. The histopathological examination may identify granuloma with caseating necrosis, Langerhans giant cells, conglomerate epithelioid histiocytes, and disproportionate submucosal inflammation [1]. Although these findings are widely described, the percentage of positive samples varies widely between centers, with a prevalence of positive results ranging from 13% to 97% [1]. The usage of the full automated real-time PCR-based test GeneXpert shows a sensitivity between 81% and 96% in the diagnosis of intestinal TB. Nonetheless, it still cannot replace the microbiological study as a gold standard test [1]. The use of IGRA is limited, even though some studies show a sensitivity between 74% and 88% and a specificity between 74% and 86%. RT-PCR using ileocecal mucosal biopsy tissue specimens and fecal specimens shows a specificity high enough to establish a diagnosis of intestinal tuberculosis but has a low sensibility [1]. On the other hand, the use of multiplex-polymerase chain reaction (multiplex-PCR) is shown to have a greater sensitivity than microbiological examination and specificity close to 100% [1]. Some immunological markers of the peripheral blood were studied in order to help distinguish between intestinal tuberculosis and other diseases. The major results found are about Forkhead box P3 (FOXP3) and CD73 [1]. However, the number of studies is minimal, and more evidence is needed [1]. Moreover, the definite diagnosis of extrapulmonary tuberculosis does not exclude concomitant pulmonary involvement since the two can coexist. Even though the diagnosis of simultaneous pulmonary infection might not change the management of extrapulmonary tuberculosis, it has an impact on the approach to contact investigations [9]. There is little information about the yield of AFB smears and mycobacterial cultures of sputum in patients diagnosed with extrapulmonary TB. According to Parimon et al., sputum smears and mycobacterial cultures may find potentially infectious cases of pulmonary TB, as was shown in a study performed in 2008, where from 57 patients diagnosed with extrapulmonary TB, AFB smears were positive in five (9%), and mycobacterial cultures were positive in 12 (21%) [9].

In the case of our patient, there were some limitations to an adequate and quick diagnosis. First, due to being a small regional hospital, there was no available Gastroenterology team to perform the colonoscopy earlier. Additionally, the absence of mycobacterial examination of the ileo/cecal biopsies, namely through acid-fast bacilli stain or RT-PCR for MTB, further delayed the diagnosis, as did the lack of identification of *Mycobacterium tuberculosis* in any of the biological samples, including sputum and bronchoalveolar lavage. Later on, IGRA was positive, adding to the suspicion of intestinal TB; however, it only confirmed early exposure to *Mycobacterium tuberculosis*. Anatomopathological examination of the cervical lymphadenopathy showed granulomatous lymphadenitis with necrosis, although when the results came out, the patient was already on antituberculostatic therapy. Again, due to the sample being insufficient for mycobacterial examination, there was no possibility of identifying *Mycobacterium tuberculosis* in it. Finally, only when lung cavitation appeared after nearly two months of therapy; it was possible to identify MTC by RT-PCR in bronchoalveolar lavage.

Patients with suspected intestinal tuberculosis should undergo an imaging examination. Computed tomography (CT)/magnetic resonance (MR) with an enterography protocol are the most recommended exams, allowing cross-sectional evaluation of intestinal tract involvement and/or other organs; of the presence of ascites, peritoneal involvement, or lymphadenopathy; and the evaluation of possible complications [10-13]. CT typically shows a concentric mural thickening in the ileocecal region, asymmetric thickening of the medial cecal wall, and lymphadenopathy with hypodense centers in the adjacent mesentery [13,14]. If the patient presents with ascites, a paracentesis is indicated. The peritoneal fluid usually shows a lymphocytic predominance with elevated Adenosine Deaminase (ADA) with values between 30-39 International Units/L. The use of ultrasound is useful for detecting lymphadenopathy, ascites, peritoneal thickening, and bowel wall thickening [15].

The treatment of intestinal tuberculosis is pharmacological and may require surgery for patients with complications. Considering the difficulty in reaching a definitive diagnosis in patients with high suspicion of infection based on clinical, epidemiological, and auxiliary diagnostic tests, the initiation of empirical therapy with tuberculostatic drugs is reasonable. The antituberculostatic therapy for intestinal tuberculosis is the same as the pulmonary variant [16]. Although fever usually resolves within one week and ascites improve within a few weeks, in 90% of the patients, the initiation of therapy may be associated with a worsening of the strictures due to scar tissue formation [17,18]. The absence of a clinical response within four to eight weeks of treatment should lead to an exhaustive investigation to exclude other pathologies such as Crohn's disease, lymphoma, or malignancy [19]. Moreover, there is also the risk of adverse effects due to hepatotoxicity caused by antituberculostatic therapy. The liver injury caused by rifampin, isoniazid, and pyrazinamide is similar, although rifampin may be associated with a cholestatic pattern with elevations in serum bilirubin and alkaline phosphatase [20]. However, drug-induced hepatitis is always a diagnosis of exclusion, and other potential causes of abnormal liver function tests should be evaluated, like alcohol consumption, acetaminophen intake, viral hepatitis, gallstones, and biliary obstruction [20]. Asymptomatic increases in aspartate transaminase concentration aren’t uncommon and can occur in nearly 20% of patients treated with the traditional four-drug regimen, resolving spontaneously in days or weeks in most patients [20]. On the other hand, if serum bilirubin is ≥3 mg/dL or serum transaminases are more than five times the upper limit of normal (or, in individuals with symptoms of hepatitis, serum transaminases are more than three times the upper limit of normal), discontinuation of therapy is needed [20]. In cases where interruption isn’t an option (such as severe disease with progressive loss of pulmonary function or current smear-positive disease), three drugs not associated with hepatotoxicity may be used until the transaminase concentrations return to lower levels [20]. The resumption of treatment after the normalization of transaminase concentrations should be performed by restarting the hepatotoxic drugs one at a time, with close monitoring between the recommencement of each agent [20].

In retrospect, considering the symptoms and epidemiological factors of our patient, he would have been a candidate for early initiation of antituberculostatic therapy. Nevertheless, the initial suspicion of inflammatory bowel disease *versus* intestinal neoplasm, based on clinical symptoms and colonoscopy findings, led to a delay in considering intestinal tuberculosis a possible cause and starting adequate treatment. On the other side, after antituberculostatic therapy was started, the patient showed significant signs of clinical and laboratory improvement. Concerning drug-induced hepatitis that prompted therapy interruption, although abdominal ultrasound and CT showed abnormalities of the liver and biliary tree, the fact that the patient also had untreated HCV infection with severe liver scarring might have contributed to the increase in the risk for toxicity.

Mortality associated with intestinal TB ranges between 1.4% and 20% [3]. High mortality rates are associated with advanced age, delay in initiating therapy, and underlying cirrhosis [4,5], as well as cases with local complications such as intestinal stricture, obstruction, perforation, and bleeding [1}.

## Conclusions

Intestinal tuberculosis is an uncommon condition in developed countries that manifests itself with symptoms that mimic an array of intestinal pathologies and thus have a wide differential diagnosis. These symptoms, aided by epidemiological risk factors and complementary exams, are key in suspecting this clinical entity. Timely diagnosis and treatment are associated with lower mortality rates.
